# Two steps forward, one step back: current harm reduction policy and politics in the United States

**DOI:** 10.1186/s12954-017-0157-y

**Published:** 2017-06-12

**Authors:** Ethan Nadelmann, Lindsay LaSalle

**Affiliations:** Drug Policy Alliance, 1330 Broadway, Suite 1426, Oakland, CA 94612 USA

**Keywords:** United States, Policies, Obama, Trump, Opioid, Overdose, Treatment, Consumption, Cannabis, Enforcement

## Abstract

Harm reduction policies and attitudes in the United States have advanced substantially in recent years but still lag behind more advanced jurisdictions in Europe and elsewhere. The Obama administration, particularly in its last years, embraced some harm reduction policies that had been rejected by previous administrations but shied away from more cutting edge interventions like supervised consumption sites and heroin-assisted treatment. The Trump administration will undermine some of the progress made to date but significant state and local control over drug policies in the US, as well as growing Republican support for pragmatic drug policies, motivated in part by the opioid crisis, ensures continuing progress for harm reduction.

## Background

Even as the United States emerged as the global pioneer in legalizing and regulating cannabis, it lags well behind much of western Europe and other regions in embracing harm reduction policies regarding other illicit drugs. Policies vary greatly among US states and even among cities within the same state, thereby making it difficult to generalize about the country as a whole, but some trends are apparent: spreading support for legalizing syringe access, even in relatively conservative parts of the country; rapid expansion of programs and policies to reduce overdose fatalities; growing law enforcement interest in harm reduction approaches to policing drug users and markets; and, belatedly, support for initiating legal drug consumption rooms in a few of the more politically progressive cities. These encouraging developments, it must be stressed, have occurred in a country in which the drug war lumbers on notwithstanding widespread disillusionment with its persistent failures, and that mostly lacks the sorts of social safety nets that cushion the harms of drug misuse and prohibitionist policies in other economically advanced nations.

The Obama administration, particularly during its last years, embraced elements of harm reduction that had been rejected by the Reagan, Clinton, and both Bush administrations. Republican and other conservative politicians also demonstrated a new openness, driven to some extent by rapidly growing opioid addiction problems among their disproportionately white constituents. The Trump administration can be counted on to undermine some of the progress made to date but there is only so much harm it can do given growing Republican support for pragmatic drug policies as well as significant state and local control in this domain.

### Syringe exchange

The great shame and tragedy of the US response to HIV/AIDS among people who inject drugs has been the persistent resistance to allowing and facilitating legal access to sterile syringes. Even the most liberal jurisdictions lagged behind much of Europe and Australia in embracing syringe exchange policies in the 1980s and 1990s, while more conservative parts of the country still prohibit such programs [1, 2]. Fourteen states today have no syringe exchange program (SEP); in 12 others, programs are only available in one or two cities [3]. The programs that do exist meet only three percent of the estimated need [4]. Countless people have died of HIV/AIDS who would likely have survived had the US implemented these programs when other fast acting nations did.

There has, however, been notable progress—at least by US standards. SEPs now operate in 196 cities, primarily along the east and west coasts [5]. HIV diagnoses among injection drug users declined by 70% in the 10-year period from 2002 to 2011, in good part because of increased access to sterile syringes [4]. SEPs are now supported by leading US governmental agencies and medical and public health associations [4]. The Obama administration was openly supportive [6, 7]. And in 2015 Congress, with Republicans in control of both legislative bodies, lifted the decades-long ban^1^ on federal funding for programs that provide sterile syringes (although federal funds still may not to be used specifically to purchase syringes) [1].

The quasi-conversion of Vice President Mike Pence offers some hope that the Trump administration will not reverse recent progress. Pence had staunchly opposed SEPs both while serving in Congress from 2001 to 2010 and during his first years as Governor of Indiana. But in 2015 he was compelled to declare a public health emergency in response to an explosion of injection-related HIV cases in a rural community [8], and persuaded by public health and local law enforcement officials—as well as evidence and prayer—to temporarily authorize (albeit with no funding) SEPs [9]. In neighboring Kentucky, conservative legislators reversed their longstanding opposition to SEPs when confronted with the highest rate of Hepatitis C Virus in the country [4]. And in early 2016, the conservative Republican governor of Florida, Rick Scott, similarly signed into law a SEP bill approved by the Republican-dominated legislature when confronted with the highest rate of new HIV infections in the country [10].

It is heartening, of course, that many elected officials who once opposed SEPs have now embraced them as a pragmatic and necessary intervention. But the fact remains that countless lives were lost that could have otherwise been saved. And, of course, many more lives remain at risk due to a lingering unwillingness by some politicians to do what is needed to stem the spread of HIV, Hepatitis C, and other infectious diseases.

### Opioid agonist therapy

Opioid agonist therapy—the treatment of opioid dependence with agonist medications such as methadone and buprenorphine (Suboxone™)—is widely recognized as the most effective treatment for opioid use disorder [11]. A prevailing abstinence-only ideology coupled with a fundamental misunderstanding of these medications—notably, that they substitute “one drug for another”—has impeded full acceptance of this treatment modality in the US. With few exceptions, and unlike in dozens of other countries, methadone is only available for addiction treatment through a highly regulated and widely stigmatized system of “opioid treatment programs” which typically require patients to appear daily at specialized clinics [12, 13]. Buprenorphine, by contrast, can be prescribed or dispensed since 2002 in physician offices. It has largely avoided the popular stigma associated with methadone, no doubt in part because buprenorphine patients are more likely than methadone patients to be white, employed, and college-educated [14].

Governmental support for opioid agonist therapy has never been better [15]. In February 2015, the principal federal substance abuse agency announced that it would no longer provide federal funding to drug courts that deny agonist medications to participants under the care of a physician [16]. Later that year, President Obama issued a Presidential Memorandum directing federal agencies to conduct a review to identify barriers to treatment with medications and develop action plans to address these barriers [17]. Tremendous hurdles, however, remain. Restrictions on methadone clinics can border on the absurd. Access to buprenorphine is still limited by lack of insurance coverage as well as limitations on the number of patients that can be prescribed buprenorphine by any one provider. Patients are typically deprived of their medication if they are incarcerated [18]. Few local jails, and even fewer state prisons, allow medications to treat opioid use disorder—although Rhode Island [19], Vermont [20], and Connecticut [20] appear to be making progress. The overall result is that less than 10% of individuals with opioid dependence receive methadone [21], and only 9% of drug treatment facilities in the US offer specialized treatment of opioid dependence with opioid agonist therapy [22].

### Overdose prevention

The US is, according to the Centers for Disease Control and Prevention, in the midst of the “worst drug overdose epidemic in history [23].” In 2015, drug overdoses accounted for over 52,000 US deaths, including over 33,000—the most ever—from misuse of opioids, typically in combination with other drugs [24]. Governmental responses have been mixed, with far too much emphasis on punitive, supply reduction strategies such as prescription drug monitoring programs, crackdowns on “pill mills” and physicians specializing in pain management, increased penalties for the use and sale of opioids, particularly fentanyl, and prosecutions of people for accidental “drug-induced” homicides. There is little evidence that these approaches have reduced opioid misuse or overdose.

On the other hand, reformers, including the Drug Policy Alliance, have played a key role in advancing harm reduction interventions as an alternative response to the opioid crisis. Virtually every state in the US has passed legislation designed to improve access to the overdose antidote naloxone [25], including 44 states where it is now permitted by law for pharmacies to offer naloxone without a prescription [26], and 36 states and the District of Columbia have passed “Good Samaritan” laws, which offer protection to those calling for help during an overdose [25]. Federal agencies are following suit, and local law enforcement officials now say they would rather administer naloxone than “handcuff a corpse.” In 2014, the Department of Justice released a toolkit for law enforcement on proper use of naloxone [27]. In 2015, the federal substance abuse agency clarified that states may purchase and distribute naloxone with federal funds [28]. And both of President Obama’s drug czars, Gil Kerlikowske and Michael Botticelli, proselytized for expanded use and availability of naloxone—albeit with too much emphasis on increasing utilization by law enforcement and first responders and too little on making it more available to community-based organizations with the greatest potential to distribute it widely and efficiently to people who use drugs.

The growing support for overdose prevention efforts, not unlike the recent spurt in Republican support for SEPs, can be explained not just by the magnitude of the crisis but also by the perception of who is most affected. Where past opioid epidemics were seen primarily in terms of inner city African Americans getting addicted to heroin, the current epidemic is perceived, more correctly than not [29], as disproportionately affecting white, middle class people getting addicted to pharmaceutical opioids [30]. The result, *The New York Times* has noted, is a “gentler drug war [31].” Republican legislators who long championed drug war policies now propose more humane responses. The Comprehensive Addiction and Recovery Act, passed by a conservative Congress in 2016, for instance, advanced naloxone and opioid agonist therapy among other interventions; Congress subsequently approved $1 billion in new federal funding in the 21st Century Cures Act for states to fight opioid misuse, including expanding access to treatment programs [32, 33]. During the 2016 presidential primaries, many of the Republican candidates, including Donald Trump, were taken aback by the frequency and urgency of pleas for help from mostly white voters.

The healthcare reform advanced by President Trump and Republican leaders in Congress in March 2017 would have significantly restricted access to treatment for people struggling with opioid addiction [34], but ultimately failed to move forward. It remains to be seen how future efforts will affect treatment coverage and options. Donald Trump is keenly aware that “his people” expect him to do something, which is why he appointed one of his most trusted advisors, Kellyanne Conway, to address the issue [35] and has also recently created an opioid commission as part of the new White House Office of American Innovation, led by Jared Kushner, Trump’s son-in-law and senior adviser [36]. Trump also appointed New Jersey Governor Chris Christie to chair the commission who, in contrast to drug war zealot US Attorney General Jeff Sessions, has emphasized addiction treatment and overdose prevention (notwithstanding his often punitive approach to drug policy more generally) [37]. The Sessions-Christie juxtaposition signals Trump’s need to appeal broadly to both law-and-order conservatives as well as the white working class constituents who seek a more compassionate approach to addressing opioid misuse [37].

### Supervised consumption sites

Supervised consumption sites, also known as safe injection facilities or drug consumption rooms, are legally sanctioned programs that allow people to consume illicit drugs under the supervision of trained staff. Overwhelming evidence demonstrates that such sites minimize the risk of infectious disease transmission, eliminate overdose fatalities, reduce public nuisance and increase referral to drug treatment and other health services [38]. They first began to open in Europe in the 1980s; today, approximately one hundred sites operate in 66 cities in Europe, Canada and Australia—but not yet in the US [39, 40].

Support for opening supervised consumption sites has grown rapidly, however, in recent years, with harm reduction and drug policy reform advocates providing much of the impetus, and the opioid crisis providing a sense of urgency. State legislators in California [41], Maryland [42], Massachusetts [43], and Vermont [44] have introduced bills to establish such sites, and the City of Ithaca, New York attracted national attention [45, 46] by including a proposal for a site in their widely-publicized municipal drug strategy [47]. The betting, though, is on Seattle, Washington, where the King County Board of Health recently voted unanimously [48] to establish two pilot consumption sites in the Seattle region [49]. The biggest obstacle is the prospect of federal intervention;^2^ a local Republican legislator recently wrote to Attorney General Sessions—a true “drug war dinosaur”—asking him to block the initiative on the grounds that it violates federal law [50]. It is hard to be optimistic at this point given the drug war proclivities of the Trump administration, but progressive drug policy has always been made at the state and local level first, and often even in the face of federal obstacles.

### Heroin-assisted treatment

Heroin-assisted treatment (HAT), or heroin maintenance, involves providing pharmaceutical-grade heroin, in specialized clinics under medical supervision, to people who would like to stop using illicit heroin but have been unable to do so with conventional treatment methods. Heroin maintenance is currently available in Switzerland, the Netherlands, Germany, Denmark, and Canada with additional trial programs having been completed in the UK, Spain and Belgium [51, 52]. In 1999, the North American Opiate Medication Initiative (NAOMI) initially included plans for three sites in the US as part of a multi-site randomized controlled trial of HAT but regulatory barriers, scant hope of government funding, and other obstacles blocked progress then and in the years since; the trial ultimately proceeded, successfully, in Canada alone [53].

Only in the past two years has interest emerged, largely as a result of advocacy by the Drug Policy Alliance. Legislators in Maryland [54] and Nevada [55] have introduced bills to permit (and, in the case of Nevada, fund) HAT pilot projects. In New Mexico—often the first state to advance novel harm reduction approaches—the legislature convened a joint committee hearing to query experts about this treatment [56]. In June 2017, the Drug Policy Alliance will convene researchers in an effort to advance prospects of a HAT research trial in the US. Though the current political climate is challenging, we are hopeful that a tightly designed research protocol can pass muster with the primary regulatory agencies and that private funding can be secured to pay for a trial. It is worth noting that US-based researchers do currently import pharmaceutical heroin, with the approval of the Drug Enforcement Administration, to administer to human subjects, albeit not for HAT [57].

Numerous US researchers have suggested that efforts focus on injectable hydromorphone (Dilaudid™) rather than heroin^3^ in order to reduce both the red tape associated with researching Schedule 1 controlled substances and the kneejerk responses to be expected by proposals to give legal heroin to people addicted to heroin. We believe, however, that initial efforts should focus on advancing HAT given the potential public education benefits, and view hydromorphone as a fallback option if the political obstacles prove too severe.

### Law Enforcement Assisted Diversion

Frustrated by the persistent failure and costs of traditional law enforcement approaches to illicit drug use, political and law enforcement officials in US cities are now examining and implementing Law Enforcement Assisted Diversion (LEAD) programs. LEAD is a pre-booking diversion program that immediately redirects people committing low-level drug offenses into community-based support services—housing, healthcare, drug treatment, and mental health services—instead of processing them through the criminal justice system [58]. Seattle, Washington was the first to roll out a LEAD program; Santa Fe, New Mexico followed shortly thereafter, extending the program to more rural environs. Initial evaluations have been strongly positive [59–61]. With interest growing nationally, the Obama administration convened law enforcement and other officials from over 30 cities, counties and states in July 2015 to learn more about LEAD [62]. The Trump administration will likely prove less enthusiastic but LEAD programs are now emerging, always with local law enforcement support, in dozens of jurisdictions, including politically conservative localities like Atlanta, Georgia, Louisville, Kentucky, and Fayetteville, North Carolina that historically have been averse to harm reduction policies [63].

Key to the success of LEAD is its attraction to police and prosecutors who appreciate the extent to which it affords them both substantial discretion and viable options in dealing with people caught in possession of illicit drugs for personal use. Labeling as “harm reduction” any intervention that relies first and foremost on police interventions is of course controversial but LEAD does appear to advance many of the same objectives as traditional harm reduction programs. It can reasonably be depicted as either the least coercive form of drug enforcement or the closest thing to Portugal-style decriminalization in the US. As LEAD becomes more widespread, vigilance will be essential to ensure that its originating harm reduction values persist and flourish.

### Cannabis

No overview of harm reduction in the United States would be complete without recognizing the global leadership of US states in legalizing medical and recreational cannabis. Legalization, i.e., legal regulation, represents the ultimate form of harm reduction given the extent to which it eliminates the many negative consequences of cannabis criminalization for consumers. The harm reduction benefits of cannabis legalization, however, extend to those involved with other drugs as well.

Abundant evidence is emerging of people in pain turning to cannabis rather than opioids, with significant benefits to health including apparent reductions in mortality. Eighty percent of medical cannabis users, in one recent study, reported substituting cannabis for prescribed medications, particularly among patients with pain-related conditions [64]. Cannabis treatment “may allow for opioid treatment at lower doses with fewer [patient] side effects,” concluded another study [65]. Yet another, in *JAMA*, found that “[s]tates with medical cannabis laws had a 24.8% lower mean annual opioid overdose mortality rate compared with states without medical cannabis laws [66].” “States permitting medical cannabis dispensaries,” a RAND BING Center for Health Economics working paper observed, “experienced a 15 to 35 percent decrease in substance abuse admissions and opiate overdose deaths [67].” Some doctors, and even rehabilitation centers, are recommending medical cannabis as a means of helping their patients use less, or abstain entirely, from opioids [68, 69]—although research is so far inconclusive on the potential of cannabis to treat opioid addiction [70].

Elected officials, including Senator Elizabeth Warren as well as the outstanding advocate on Capitol Hill for cannabis reform, Congressman Earl Blumenauer, are now speaking publicly about this evidence [71]. President Trump spoke favorably of medical cannabis during his campaign, and his press secretary, Sean Spicer, reaffirmed this support in late February [72]. Attorney General Sessions, however, has provided little reassurance while mocking the notion that cannabis can play a constructive role in ameliorating the opioid crisis [73]. The Trump administration can do little at this point to stem the growing support for cannabis legalization, which now approaches 60% [74], but it will try to slow the implementation and expansion of legal regulation by state governments [75].

## Conclusion

Ideological resistance to harm reduction is fading, albeit far too slowly, in the US. The most politically progressive cities and states are increasingly supportive, even as they lag ten to twenty years behind leading cities in Europe, Australia, and Canada in implementing supervised consumption sites and heroin-assisted treatment. More conservative jurisdictions, and their elected officials, are beginning to accept syringe exchange programs and the value of opioid agonist therapy. Abstinence-only treatment programs still dominate but harm reduction precepts and practices are proliferating as a new generation of treatment professionals assert their influence [76]. The opioid epidemic in the US is forcing much of this new openness, especially in more conservative parts of the country, as mostly Republican-elected officials struggle to respond to cries for help by desperate, disproportionately white, constituents.

It is hard to be hopeful about anything involving the Trump administration. Federal funding for research, harm reduction, and treatment services will likely decline. Federal authorities may attempt to block municipal initiatives to open supervised consumption sites. President Trump and his attorney general seem far more eager to stir up fears about violent crime than to embrace the benefits of LEAD programs. The modest but historically significant reforms on international drug policy embraced by the Obama administration in recent years may not survive. But the benefits of harm reduction—notably its public health and fiscal advantages—in addressing the opioid crisis cannot be overestimated. And, given how much harm reduction policies and attitudes are now embedded in state and local governance, they will surely outlast the Trump administration.

## Abbreviations

HAT: Heroin-assisted treatment
HIV/AIDS: Human immunodeficiency virus/acquired immune deficiency syndrome
LEAD: Law Enforcement Assisted Diversion
SEP: Syringe exchange program

## Footnotes

1 The ban was briefly lifted, with President Obama’s support, by the Democratic-controlled Congress in 2009 after being in place for more than 20 years. Republicans restored the ban in 2011 after regaining control of Congress.

2 Though the federal government has attempted to create barriers to state-led efforts to provide access to sterile syringes, possession of syringes is not illegal under federal law. Concerns about potential federal enforcement or federal preemption were accordingly minimal. Supervised consumption sites, on the other hand, permit the possession and consumption of controlled substances and so arguably run afoul to federal law.

3 The “Study to Assess Longer-term Opioid Medication Effectiveness” in Canada compared two injectable medications – diacetylmorphine, the active ingredient of heroin, and hydromorphone, a legal, licensed pain medication – and found that hydromorphone was as effective as diacetylmorphine for long-term opioid dependence [77].

## References

[1] Weinmeyer R (2016). Needle exchange programs’ status in US politics. Am Med Assoc J Ethics.

[2] Bassler, SE. The history of needle exchange programs in the United States. In: Master’s and doctoral projects. University of Toledo direct repository. 2007. Paper 275, http://utdr.utoledo.edu/graduate-projects/275. Accessed 13 Mar 2017.

[3] North American Syringe Exchange Network. Directory of syringe exchange programs. https://nasen.org/directory/. Accessed 13 Mar 2017.

[4] Hodel D (2015). Preventing HIV and Hepatitis C among people who inject drugs: public funding for syringe services, programs makes the difference. amFAR Issue Brief.

[5] The Foundation for AIDS Research (amFAR). Syringe service program coverage in the United States: June 2014. In: syringe service programs. 2014. https://nasen.org/site_media/files/amfar-sep-map/amfar-sep-map-2014.pdf. Accessed 13 Mar 2017

[6] White House Office of National Drug Control Policy, Senate Judiciary Committee confirmation hearing, 111^th^ Congress. Statement of Gil Kerlikowske, Director of ONDCP. 2009.

[7] Schreiner B. McConnell, drug czar talk heroin in N. Ky. In: associated press. Courier Journal. 2015. http://www.courier-journal.com/story/news/local/2015/04/09/mcconnell-botticelli/25544135/. Accessed 14 Mar 2017.

[8] HIV outbreak in southeastern Indiana. In: Indiana State Department of Health. Indiana Gov. 2015. http://www.in.gov/isdh/26649.htm. Accessed 14 Mar 2017.

[9] Twohey M. Mike Pence’s response to H.I.V Outbreak. In: Election 2016. New York Times. 2016. https://www.nytimes.com/2016/08/08/us/politics/mike-pence-needle-exchanges-indiana.html?_r=1. Accessed 27 Mar 2017

[10] Auslen M. Gov Rick Scott signs needle exchange, rape kit bills into law. Miami Dade County. Miami Herald. 2016. http://www.miamiherald.com/news/local/community/miami-dade/article67779477.html. Accessed 27 Mar 2017.

[11] National consensus development panel on effective medical treatment of opiate addiction (1998). Effective medical treatment of opiate addiction. JAMA.

[12] Rettig RA, Yarmolinsky A (1995). Federal regulation of methadone treatment. Inst Med Natl Acad Press.

[13] Kleber HD (2008). Methadone maintenance four decades later: thousands of lives saved but still controversial. JAMA.

[14] Hansen HB, Siegel CE, Case BG (2013). Variation in use of buprenorphine and methadone treatment by racial, ethnic and income characteristics of residential social areas in New York City. J Behav Health Serv Res.

[15] The White House Press Secretary. National Drug Control Strategy. The White House of President Barack Obama. 2015. page 29, https://obamawhitehouse.archives.gov/ondcp/policy-and-research/ndcs. Accessed 27 Mar 2017.

[16] Knopf A. SAMHSA bans drug court grantees from ordering participants off MAT. In: Alcoholism and Drug Abuse Weekly. Jossey-Bass. 2015. http://www.alcoholismdrugabuseweekly.com/Article-Detail/samhsa-bans-drug-court-grantees-from-ordering-participants-off-mat.aspx. Accessed 27 Mar 2017

[17] White House Press Secretary. Obama administration announces public and private sector efforts to address prescription drug abuse and heroin use. In: statements and releases. The White House of President Barack Obama. 2015. https://www.whitehouse.gov/the-press-office/2015/10/21/fact-sheet-obama-administration-announces-public-and-private-sector. Accessed 27 Mar 2017.

[18] Rich JD, Boutwell AE, Shield DC (2005). Attitudes and practices regarding the use of methadone in US state and federal prisons. J Urban Health.

[19] Kirsch N. R.I.’s new medication assisted treatment program for drug addicted inmates praised. In: health care. Providence Business News. 2016. http://pbn.com/RIs-new-medication-assisted-treatment-program-for-drug-addicted-inmates-praised,116053?print=1. Accessed 27 Mar 2017.

[20] UMass medical school partnering with Connecticut, Massachusetts, and Rhode Island to improve treatment of substance use disorder in prison populations. In: Commonwealth Medicine. University of Massachusetts Medical School. 2017. https://commed.umassmed.edu/news/2017/01/31/umass-medical-school-partnering-connecticut-massachusetts-and-rhode-island-improve. Accessed 27 Mar 2017.

[21] Nosyk B, Anglin D, Brissette S (2015). A call for evidence-based medical treatment of opioid dependence in the United States and Canada. Health Aff.

[22] Substance Abuse and Mental Health Services Administration. National Survey of Substance Abuse Treatment Services. 2011 Data. Programs or Groups for Specific Client Types –Table 4.11b. 2012. http://www.samhsa.gov/data/DASIS/2k11nssats/NSSATS2011Chp4.htm. Accessed 28 Mar 2017.

[23] Paulozzi L. The epidemiology of fatal drug overdoses: potential for prevention. Presented at Promising Legal Responses to the Epidemic of Prescription Drug Overdoses in the United States. Atlanta. 2008.

[24] Rudd R, Seth P, David F, Scholl L. Increases in drug and opioid overdose deaths — United States, 2010–2015. Morbidity and Mortality Weekly Report, CDC. 2016; 65.50-51: 1445–1452. https://www.cdc.gov/mmwr/volumes/65/wr/mm655051e1.htm. Accessed 28 Mar 2017. 10.15585/mmwr.mm655051e1 28033313

[25] Davis C, Chang S, Carr D. Legal interventions to reduce overdose mortality: naloxone access and overdose good samaritan laws. The Network for Public Health Law. 2016. https://www.networkforphl.org/_asset/qz5pvn/network-naloxone-10-4.pdf. Accessed 28 Mar 2017

[26] Davis C, Carr D (2017). State legal innovations to encourage naloxone dispensing. J Am Pharm Assoc.

[27] Law enforcement and naloxone. In: naloxone toolkit content. Bureau of Justice Assistance. https://www.bjatraining.org/tools/naloxone/Naloxone%2BBackground. Accessed 28 Mar 2017.

[28] Substance Abuse and Mental Health Services Administration. Expansion of naloxone in the prevention of opioid overdose FAQs. https://www.samhsa.gov/sites/default/files/programs_campaigns/medication_assisted/expansion-of-naloxone-faq.pdf. Accessed 13 Mar 2017.

[29] The Henry J. Kaiser Family Foundation. Opioid overdose deaths by race/ethnicity. 2014. http://kff.org/other/state-indicator/opioid-overdose-deaths-by-raceethnicity/?currentTimeframe=0&sortModel=%7B%22colId%22:%22Location%22,%22sort%22:%22asc%22%7D. Accessed 30 Mar 2017

[30] Netherland J, Hansen H (2016). White opioids: pharmaceutical race and the war on drugs that wasn’t. BioSocieties.

[31] Seelye K. In heroin crisis, white families seek gentler war on drugs. The New York Times. 2015. http://www.nytimes.com/2015/10/31/us/heroin-war-on-drugs-parents.html?_r=0. Accessed 27 Mar 2017

[32] Drug Policy Alliance. Congress passes landmark opioid bill - the Comprehensive Addiction and Recovery Act (CARA). press release. 2016. http://www.drugpolicy.org/news/2016/07/congress-passes-landmark-opioid-bill-comprehensive-addiction-and-recovery-act-cara. Accessed 27 Mar 2017.

[33] Steinhauer J, Tavernise S. $6.3 billion measure aims to cure ailing health care policies. In: politics. The New York Times. 2016. https://www.nytimes.com/2016/11/28/us/politics/congress-cures-cancer-moonshot-alzheimers.html. Accessed 29 Mar 2017.

[34] Seelye K, Goodnough A. Addiction treatment grew under health law. In: health. New York Times. 2017. https://www.nytimes.com/2017/02/10/health/addiction-treatment-opiods-aca-obamacare.html?_r=0. Accessed 27 Mar 2017.

[35] Palmeri T. Kellyanne Conway’s dangerous game. In: white house. Politico. 2017. http://www.politico.com/story/2017/02/kellyanne-conways-dangerous-game-234935. Accessed 27 Mar 2017.

[36] Rucker P. Chris Christie to lead Trump White House drug commission. In: politics. Washington Post. 2017. https://www.washingtonpost.com/news/post-politics/wp/2017/03/29/chris-christie-to-lead-trump-white-house-drug-commission/?utm_term=.a9e2e6c2a4fa. Accessed 27 Mar 2017.

[37] Reinhard B. Trump’s drug policy takes shape, with split personality. In: politics. Wall Street Journal. 2017. https://www.wsj.com/articles/trumps-drug-policy-takes-shape-with-split-personality-1490952605. Accessed 03 Apr 2017.

[38] Potier C, Laprevote V, Dubois-Arber F, Cottencin O, Rolland B (2014). Supervised injection services: what has been demonstrated? A systematic literature review. Drug Alcohol Depend.

[39] Schatz E, Nougier M. Drug consumption rooms: evidence and practice. International Drug Policy Consortium. 2012. http://idpc.net/publications/2012/06/idpc-briefing-paper-drug-consumption-roomsevidence-and-practice. Accessed 27 Mar 2017

[40] Drug consumption rooms: an overview of provision and evidence. European Monitoring Centre for Drugs and Drug Addiction. 2016. http://www.emcdda.europa.eu/topics/pods/drug-consumption-rooms. Accessed 27 Mar 2017.

[41] An act to add section 11376.6 to the health and safety code, relating to controlled substances. AB 186. California State Assembly. 2017. http://leginfo.legislature.ca.gov/faces/billNavClient.xhtml?bill_id=201720180AB186. Accessed 27 Mar 2018.

[42] Public health – overdose and infectious disease prevention safer drug use facility program. HB 519. Maryland State Legislature: House. 2017. http://mgaleg.maryland.gov/2017RS/bills/hb/hb0519f.pdf. Accessed 28 Mar 2017.

[43] An act to authorize public health workers to pursue new measures to reduce harm and stigma for people affected by substance use disorder. S 1081. Massachusetts State Senate. (2017). https://malegislature.gov/Bills/190/SD1775. Accessed 13 Mar 2017.

[44] An act relating to limiting drug-related criminal liability and civil forfeiture actions against persons associated with an approved safer drug consumption program. H108. Vermont State Legislature: House. (2017). http://legislature.vermont.gov/assets/Documents/2018/Docs/BILLS/H-0108/H-0108%20As%20Introduced.pdf. Accessed 28 Mar 2017.

[45] Foderaro L. Ithaca’s anti-heroin plan: open a site to shoot heroin. The New York Times. 2016. https://www.nytimes.com/2016/03/23/nyregion/fighting-heroin-ithaca-looks-to-injection-centers.html?_r=0. Accessed 03 Apr 2017.

[46] Beller S. CNN’s Fareed Zakaria uses proposed safe injection facility to teach ‘NY values’ to Ted Cruz. The Influencer. http://theinfluence.org/watch-cnns-fareed-zakaria-uses-proposed-safe-injection-facility-to-teach-ny-values-to-ted-cruz/. Accessed 03 Apr 2017.

[47] The Ithaca plan: a public health and safety approach to drugs and drug policy. City of Ithaca, NY. 2016. http://www.cityofithaca.org/DocumentCenter/View/4224. Accessed 27 Mar 2017.

[48] Zezima K. Awash in overdoses, Seattle creates safe sites for addicts to inject illegal drugs. In: politics. The Washington Post. 2017. https://www.washingtonpost.com/politics/awash-in-overdoses-seattle-creates-safe-sites-for-addicts-to-inject-illegal-drugs/2017/01/27/ddc58842-e415-11e6-ba11-63c4b4fb5a63_story.html?utm_term=.592fe985784e. Accessed 27 Mar 2017.

[49] King County heroin and prescription opiate addiction task force. Final report and recommendations. In: documents. 2016. http://www.kingcounty.gov/~/media/depts/community-human-services/behavioral-health/documents/herointf/Final-Heroin-Opiate-Addiction-Task-_Force-Report.ashx?la=en. Accessed 27 Mar 2017.

[50] Jaywork C. State republican asks DoJ to stop Seattle’s planned safe drug site. In: news. Seattle Weekly. 2017. http://www.seattleweekly.com/news/state-republican-asks-doj-to-stop-seattles-planned-safe-drug-site/. Accessed 27 Mar 2017.

[51] Strang J, Groshkova T, Metrebian N (2012). New heroin-assisted treatment: recent evidence and current practices of supervised injectable heroin treatment in Europe and beyond.

[52] Health Canada to propose regulatory change to enable consideration of applications under the Special Access Programme to facilitate treatment of chronic relapsing opioid dependence. In: news releases. Government of Canada. 2016. http://news.gc.ca/web/article-en.do?mthd=tp&crtr.page=1&nid=1065009&crtr.tp1D=1. Accessed 27 Mar 2017.

[53] Gartry CC, Oviedo-Joekes E, Laliberté N (2009). NAOMI: the trials and tribulations of implementing a heroin assisted treatment study in North America. Harm Reduct J.

[54] An act relating to public safety. SB 181. 79th State Senate. 2017. http://www.leg.state.nv.us/Session/79th2017/Bills/SB/SB181.pdf.

[55] Poly-Morphone-Assisted Treatment Pilot Program - Harm Reduction Act 2016. House Bill 1267. Maryland House of Delegates. 2016. https://legiscan.com/MD/text/HB1267/2016. Accessed 27 Mar 2017

[56] Drug Policy Alliance. New Mexico legislative committees to hear about heroin assisted treatment. In: press release. 2016. http://www.drugpolicy.org/news/2016/10/friday-new-mexico-legislative-committees-hear-about-heroin-assisted-treatment. Accessed 28 Mar 2017.

[57] Szalavitz M (2015). Teaching recovery. Unbroken brain: a revolutionary new way of understanding addiction.

[58] Drug Policy Alliance. Law enforcement assisted diversion (LEAD): reducing the role of criminalization in local drug control. 2016. http://www.drugpolicy.org/resource/law-enforcement-assisted-diversion-lead-reducing-role-criminalization-local-drug-control

[59] Collins S, Lonczak H, Clifasefi S. LEAD program evaluation: recidivism report. Harm Reduction Research and Treatment Lab, University of Washington – Harborview Medical Center. 2015. http://static1.1.sqspcdn.com/static/f/1185392/26121870/1428513375150/LEAD_EVALUATION_4-7-15.pdf?token=6eqelJdqkm48QyPUa4BsulQh%2F9o%3D. Accessed 27 Mar 2017.

[60] Collins S, Lonczak H, Clifasefi S. LEAD program evaluation: criminal justice and legal system utilization and associated costs. Harm Reduction Research and Treatment Lab, University of Washington – Harborview Medical Center. 2015. http://static1.1.sqspcdn.com/static/f/1185392/26401889/1437170937787/June+2015+LEAD-Program-Evaluation-Criminal-Justice-and-Legal-System-Utilization-and-Associated-Costs.pdf?token=itMYMgOIGZdwDtR5Dxg7f3qTC94%3D. Accessed 17 Mar 2017.

[61] Collins S, Lonczak H, Clifasefi S. LEAD program evaluation: the impact of LEAD on housing, employment and income/benefits. Harm Reduction Research and Treatment Lab, University of Washington – Harborview Medical Center. 2016. http://static1.1.sqspcdn.com/static/f/1185392/27047605/1464389327667/housing_employment_evaluation_final.PDF?token=QMJWAbfa%2BT70A%2Fxinjxq4S9Howc%3D. Accessed 27 Mar 2017.

[62] Austin RL. LEAD-ing the way to a more efficient criminal justice system. The White House of President Barack Obama. 2015. https://www.whitehouse.gov/blog/2015/07/02/lead-ing-way-more-efficient-criminal-justice-system. Accessed 28 Mar 2017.

[63] National Support Bureau. Background. In: about the bureau. https://www.leadbureau.org/about-the-bureau. Access 27 Mar 2017.

[64] Haroutounian S, Ratz Y, Ginosar Y (2016). The effect of medicinal marijuana on pain and quality of life outcomes in chronic pain: a prospective open-label study. Clin J Pain.

[65] Abrams DI, Couey P, Shade SB (2011). Cannabinoid-opioid interaction in chronic pain. Clin Pharmacol Ther.

[66] Bachhuber MA, Saloner B (2014). Medical cannabis laws and opioid analgesic overdose mortality in the United States, 1999-2010. JAMA Intern Med.

[67] Powell D, Pacula RL, Jacobson M. Do medical marijuana laws reduce addictions and deaths related to pain killers? National Bureau of Economic Research. Working Paper No. 21345. 2015. 10.1016/j.jhealeco.2017.12.007 PMC7867411 29408153

[68] Villani C. Doctors pioneer pot as an opioid substitute. Boston Herald. 2015. http://www.bostonherald.com/news_opinion/local_coverage/2015/10/doctors_pioneer_pot_as_an_opioid_substitute. Accessed 29 Mar 2017.

[69] Porter J. Can this man successfully treat opioid addiction with marijuana? The Guardian. 2017. https://www.theguardian.com/science/2017/mar/09/opioid-addiction-marijuana-treatment-joe-schrank-high-sobriety. Accessed 28 Mar 2017.

[70] Hurd YL, Yoon M, Manini AF (2015). Early phase in the development of cannabidiol as a treatment for addiction: opioid relapse takes center stage. Ntl Center for Biotechnology Information: Neurotherapeutics.

[71] Warren E. Letter to CDC re opioid epidemic research. 2016. https://www.warren.senate.gov/files/documents/2016-2-8_Letter_to_CDC_re_opioid_epidemic%20research.pdf. Access 14 Mar 2017.

[72] Nadelmann E. As Trump said in the campaign, leave pot to the states. In: the opinion pages. The New York Times. 2017. https://www.nytimes.com/2017/02/27/opinion/as-trump-said-in-the-campaign-leave-pot-to-the-states.html. Accessed 03 Apr 2017.

[73] Sifferlin A. Jeff Sessions says marijuana is only ‘slightly less awful’ than heroin. Science says he’s wrong. Time. Health. 2017. http://time.com/4703888/jeff-sessions-marijuana-heroin-opioid/. Accessed 03 Apr 2017.

[74] Pew Research Center. Support for marijuana legalization continues to rise. 2016. http://www.pewresearch.org/fact-tank/2016/10/12/support-for-marijuana-legalization-continues-to-rise/. Accessed 03 Apr 2017.

[75] Halper E, McGreevy P. Trump administration signals a possible crackdown on states over marijuana. In: politics. The Los Angeles Times. 2017. http://www.latimes.com/politics/la-na-pol-trump-marijuana-20170223-story.html. Accessed 30 Mar 2017.

[76] Wren, C. In battle against heroin, scientists enlist heroin. The New York Times. 1999. http://www.nytimes.com/1999/06/08/science/in-battle-against-heroin-scientists-enlist-heroin.html. Accessed 27 Mar 2017

[77] Oviedo-Joekes E, Guh D, Brissette S (2016). Hydromorphone compared with diacetylmorphine for long-term opioid dependence. JAMA.

